# Cervical lymphadenopathy as initial presentation of metastatic prostate cancer: A retrospective study of five cases and literature review

**DOI:** 10.3389/fsurg.2023.1081951

**Published:** 2023-01-30

**Authors:** Yangyang Liu, Zhihong Dai, Jiange Hao, Liang Wang, Zhiyu Liu

**Affiliations:** ^1^ Department of Urology, The Second Affiliated Hospital of Dalian Medical University, Dalian, China; ^2^ Department of Emergency, Xijing Hospital in Xi’an City, Xi’an, Shanxi, China

**Keywords:** metastatic prostate cancer, supraclavicular lymphadenopathy, cervical lymphadenopathy, cervical lymph nodes, case report

## Abstract

Cervical lymphadenopathy as the initial presentation of metastatic prostate cancer is particularly uncommon, and easily misdiagnosed. In the current study, we describe five cases of metastatic prostate cancer in our hospital that presented with cervical lymphadenopathy as an initial symptom. The diagnosis was confirmed by needle biopsy of the suspicious lymph nodes and the serum prostate specific antigen (PSA) levels of all patients exceeded 100 ng/ml. The five patients were treated with hormonal therapy; four received traditional hormonal therapy, including bicalutamide and goserelin; one patient received hormonal therapy that included abiraterone and goserelin. Case 1 developed into castration-resistant prostate cancer (CRPC) after 7 months and died after 12 months. Case 2 rejected regular hormonal therapy for personal reasons and died 6 months after the initial diagnosis. Case 3 was still alive at the time of writing. Case 4 was administered with abiraterone, prednisolone and goserelin; the treatment was effective and the patient has remained symptom-free for the last 24 months. Case 5 was treated with hormonal and chemotherapy but died 8 months after diagnosis. In conclusion, any elderly male presenting with cervical lymphadenopathy should be considered the possibility of prostate cancer, especially when the needle biopsy reveals adenocarcinoma. The prognosis for patients presented with cervical lymphadenopathy as the initial presentation is usually poor. Hormone therapy based on abiraterone may yield a better response in such cases.

## Introduction

Prostate cancer is the second most common form of cancer in men (1). According to the latest data, there were 1,143,300 new cases of prostate cancer worldwide in 2020 (2). The axial skeleton and regional lymph nodes are the most common sites of metastases for prostate cancer,followed by the lungs, bladder, liver and adrenal glands (3). However, distal metastases to the cervical lymph nodes are very rare, especially as an initial presentation for prostate cancer (4), the incidence of this condition is less than 0.15% (5). Besises, the presence of metastatic lymph nodes is a poor prognostic factor in patients with prostate cancer, and the survival rates are significantly reduced in such patients (6). Herein, we report five rare cases presenting with cervical lymph node enlargement as an initial diagnosis between January 2010 and December 2021 in our hospital. We also provide a review of the current literature.

## Materials and methods

First, we gained approval from the Ethics Committee of the Second Affiliated Hospital at Dalian Medical University, and the permision for publication has been obtained from the patients or their representatives. Next, performed a retrospective review of all patients diagnosed with prostate cancer in our hospital between January 2010 and December 2021. The inclusion criteria were as follows: (1) patients admitted to our hospital with a neck mass as the initial symptom and (2) the primary site was confirmed to be the prostate. There were no restrictions with regards to the department at which the patient first presented. The exclusion criteria were as follows: (1) prostate cancer had been diagnosed in another hospital prior to attendance at our hospital; (2) there was an incomplete set of patient follow-up records. The pathological findings of the prostate biopsies were the gold standard for diagnosing prostate cancer. And the source of the cervical lymph lodes was also confirmed by the pathological outcomes of the needle biopsy. In this research study, we identified 2,248 patients who had been diagnosed with prostate cancer. Of these patients, only five males presented with enlargement of the cervical lymph nodes as the initial manifestation of metastatic prostate cancer and written informed consent was obtained from the patient.

## Results

### Basic information relating to the five cases

Of 2,248 patients, only five males presented with enlargement of the cervical lymph nodes as the initial manifestation of metastatic prostate cancer. The five patients were all transferred to the Urology Department from other clinics; four from Otorhinolaryngology and one from Oncology. The basic clinical details of these patients are given in Table 1. All five patients had cervical lymphadenopathy as the initial presentation. Four of these patients were referred to the hospital presenting with a palpable painless mass on the left cervical neck, while one patient showed bilateral neck enlargement in the lymph nodes. Mean age at the time of initial diagnosis was 65.4 ± 5.6 years. All of the patients except for case 2 denied any urinary complaints when they were firstly referred to our hospital due to cervical lymphadenopathy; case 2 reported that he found new onset gross painless hematuria, although this was not accompanied by frequent urination, urgency or pain. Case 3 had undergone bilateral partial thyroidectomy four years previously. Case 5 had undergone resection of a gastrointestinal villous tubular adenoma one year previously. The medical and family history of the other patients were unremarkable.

**Table 1:** The basic characteristics of patients with prostate carcinoma presenting with cervical lymphadenopathy.

No	Age years	Clinic in first visit	Medical history	Family history	Initial presention	Lower urinary tract symptoms	Mass diameter measured by ultrasound
1	54	Otorhinolaryngology	–	–	Thyroid nodules and mass on left neck	–	35 × 25 mm
2	67	Otorhinolaryngology	–	–	Mass on left neck	+	38 × 21 mm
3	63	Otorhinolaryngology	Thyroid carcinoma	–	Mass on bilateral neck	–	48 × 22 mm (L) 11 × 6 mm (R)
4	67	Gastrointest-inal oncology	Gastric perforation	–	Mass on left neck	–	32 × 15 mm
5	67	Otorhinolaryngology	–	–	Mass on left neck	–	15 × 10 mm

L, left; R, right.

All patients underwent regular physical and radiological examinations, although the initial diagnosis was based on the biopsy of suspicious lymph nodes or specific immunohistochemical stains. Immunohistochemical staining of the suspicious lymph nodes showed positive results for prostate-specific antigen (PSA) in all five cases, as shown in Table 2. Then, we examined tumor markers in an attempt to identify the primary lesion, thus revealing PSA level exceeding 100 ng/ml in all patients (Table 2). Except for one case that refused prostate biopsy, the remaining four patients all received ultrasound-guided transperineal treatment for prostate cancer. Pathological analysis revealed malignant prostate carcinoma; Gleason scores were 4 + 4 = 8 points, 5 + 4 = 9 points, 4 + 5 = 9 points, and 5 + 4 = 9 points, respectively. In addition, computer tomography (CT) scans of bone revealed multiple bone metastases in all five cases. CT of the chest, abdomen, and pelvis, were used to investigate the progression of local prostate tumors and the presence of systemic-organ metastases; none of the cases showed evidence of metastases in the distal organs. Furthermore, three cases underwent 18-FDG positron emission tomography computed tomography (PET CT) scans which revealed multiple lymph nodes metastases and multiple bone metastases that were not accompanied by visceral organ metastases.

**Table 2:** Pathology of neck masses and prostate tissue.

No	Needle-biopsy of the mass	PSA stain	PSA level (ng/Ml)	Prostate biopsy	Gleason score	Bone scan
1	Metastatic malignant cell	+	187.5	ND	–	+
2	Moderately differentiated adenocarcinoma	+	>10,000	PC	4 + 4 = 8	+
3	Poorly differentiated adenocarcinoma	+	323.5	PC	5 + 4 = 9	+
4	Moderately differentiated adenocarcinom	+	2296	PC	4 + 5 = 9	+
5	Poorly differentiated adenocarcinoma	+	278.8	PC	5 + 4 = 9	+

PSA, prostate specific antigen; GS score, gleason score; ND, not done; PC, prostate cancer.

### Treatments and outcomes

The five cases were initially treated with hormonal therapy (as shown in Table 3). Cases 1, 2, 3, and 5 were administered with bicalutamide and goserelin, with zoledronic acid to relieve bone complications. Case 4 was treated directly with abiraterone and goserelin directly. The serum PSA of case 1 decreased to 9.03 ng/ml after seven months of treatment; the left neck mass was significantly smaller but more accessible than before. However, Case 1 experienced significant pain symptoms; CT scanning of the bones revealed the further progression of systematic bone metastases, thus leading to the possibility castration-resistant prostate cancer (CRPC). To control disease progression, Case 1 was treated with abiraterone, but eventually died 12 months after first diagnosis due to uncontrollable gastrointestinal bleeding.

**Table 3:** Treatments and prognoses for the five cases.

No	Initial treatment	Neck mass after treatment	PSA level after 3-month treatment (ng/ml)	Time progress to CRPC	Testosterone value (nmol/L)	Treatment after CRPC	Prognosis
1	Bicameralism + goserelin + zoledronic acid	Decrease	9.03	7 months	0.15	Abiraterone + goserelin + zoledronic acid	Dead after 12 months
2	Bicameralism + goserelin + zoledronic acid	NK	–	–	NK	–	Dead after 6 months
3	Bicameralism + goserelin + zoledronic acid+	Disappeare after 4 months	0.094	12 months	0.55	Abiraterone + goserelin + zoledronic acid	18 months alive
4	Abiraterone + goserelin + zoledronic acid	Disappeared after 6 months	0.638	–	<0.07	–	24 months alive
5	Bicalutamide + goserelin + zoledronic acid+	NK	5.15	5 months	0.12	Abiraterone + prednisolone + docetaxel	Dead after 8 months

PSA, prostate specific antigen; NK, not know; CRPC, castration resistant prostate cancer.

**Figure 1:**
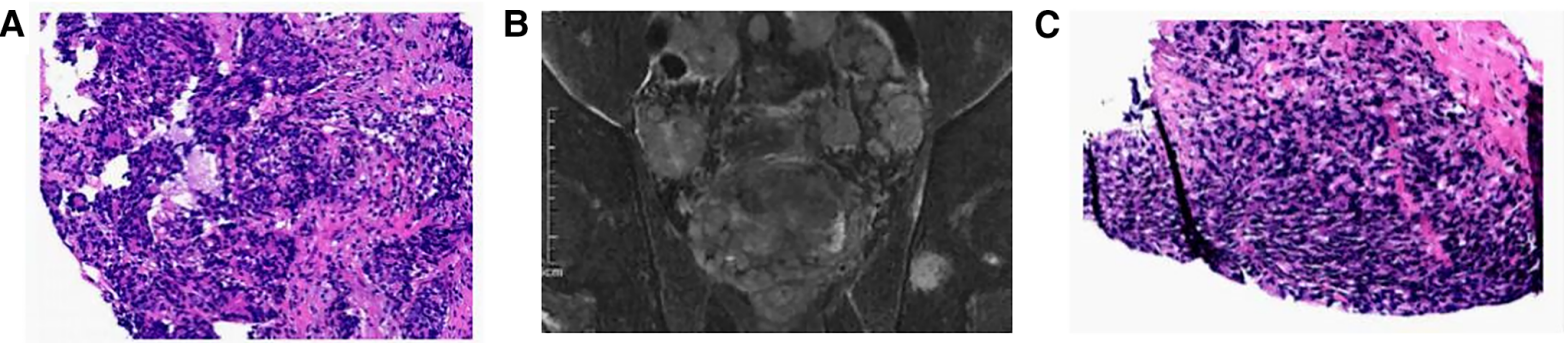
Case 4 (**A**) under the microscope, fine needle biopsy of the suspicious tissue revealed a large number of cancer cells with indistinct borders and finely granular cytoplasm. Pathology of left cervical lymph nodes revealed adenocarcinoma(HE*40). (**B**) Mp-MRI revealed prostate cancer. (**C**) Pathology of ultrasound-guided transperineal biopsy revealed malignant prostate carcinoma, Gleason score was 5 + 4 = 9 points(HE*40).

**Figure 2:**
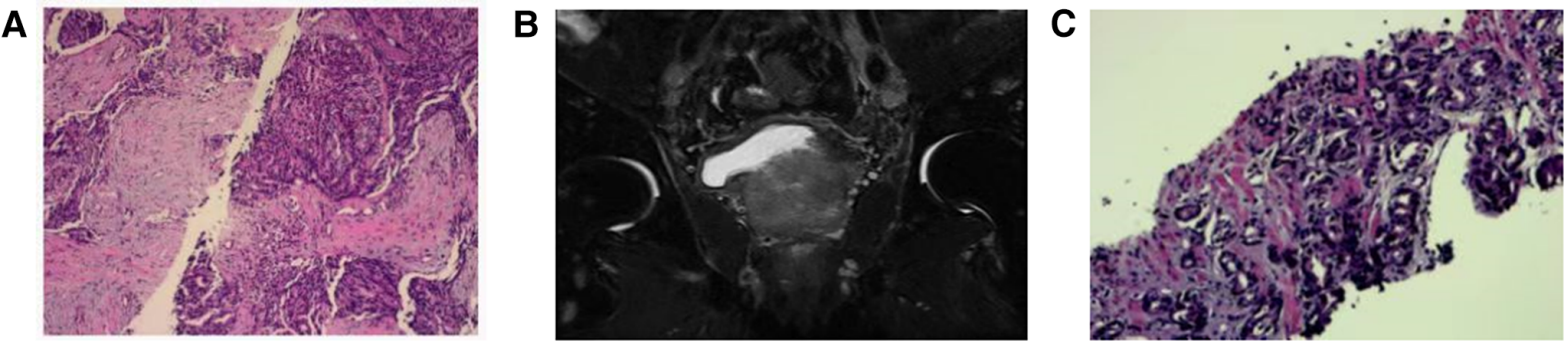
Case 5 (**A**) the left cervical lymph nodes biopsy revealed a poorly differentiated adenocarcinoma, which was considered to be based on the origin of the prostate, the immunohistochemical results were as follows: AE1/AE3(+), CK7(−), CK20(−), PSA(+), PsAP(+), NKX3.1(+), TTF1(+), TG(−), NapsinA(−), Villin(+), CDX2(−), Syn(−), CgA(−), CD56(−), Ki-67(+, 70%–80%)(HE*40). (**B**) Mp-MRI revealed prostate cancer. (**C**) Pathology of ultrasound-guided transperineal biopsy revealed malignant prostate carcinoma, Gleason score was 5 + 4 = 9 points(HE*40).

Furthermore, the prognosis of case 2 was also poor. Due to economic reasons, the patient refused to implement further treatment options and died 6 months after the initial diagnosis. Case 3 had reported beneficial effects and follow-up checks revealed that the PSA level had fallen to 0.094 ng/Ml after three months of treatment. The cervical lymph nodes had disappeared after 4 months. However, Case 3 progressed to CRPC after 12 months and abiraterone was used for the next stage of treatment. Over 18 months follow-up, the patient remained asympomatic while receiving androgen blockade. Case 4 received abiraterone, prednisolone, and goserelin; clinical follow-up 3 months later revealed a PSA of 0.638 ng/ml; the enlarged cervical lymph node also disappeared after 6 months. The patient was followed up regularly with no progression or recurrence of cancer for 24 months (Figure 1). The prognosis of case 5 was even poorer; he died 8 months after the initial diagnosis. He was initially given goserelin and bicalutamide, with zoledronic acid to target bone metastases. However, five months later, he was re-referred to the hospital due to fever and an incomplete intestinal obstruction. Upon review, the serum PSA level was 32.35 ng/ml. He received antibiotics and gastrointestinal decompression treatment for fever and incomplete intestinal obstruction, For prostate cancer, the patient was given abiraterone, goserelin and zoledronic acid. After one month of treatment, the serum PSA had increased to 59.65 ng/ml. The patient underwent 18-F PSMA PET CT scanning again; results suggested a poor response to hormone therapy and revealed that the range of bone metastases had increased when compared to previous scans; the condition of the multiple lymph nodes was similar to when assessed previously. We also observed multiple newly formed nodules in both lungs, thus excluding the possibility of metastatic cancer. The patient died after two months (Figure 2).

## Discussion

The most common metastatic sites for prostate cancer are the bones and regional lymph nodes, followed by the lungs, bladder, liver, and adrenal glands (3). Regional lymph node dissemination most frequently occurs to the obturator nodes, along with the perivesical, hypogastric, iliac, presacral, and para-aortic nodes (7). However, distal metastases of prostate cancer to the cervical lymph nodes are rare, especially when presenting as an initial symptom. Michael et al. studied approximately 6,000 patients diagnosed with prostate cancer between 1950 and 1968 at the Mayo Clinic and found that the incidence of prostate cancer metastases to supraclavicular lymph nodes, as an initial manifestation, was <0.1% (5/6000) (8), this was similar to the incidence we observed in our hospital (0.22%, 5/2248). James et al. reported 19 cases with supraclavicular lymph nodes as the initial presentation of metastatic prostate cancer (9). Our literature analysis revealed that a total of 58 cases have been reported from the 1970 s until now. Of these 58 cases, most cases of prostate cancer presented with supraclavicular lymphdenophaty involving left-side cervical lymphadenopathy; only seven patients reported have been reported with supraclavicular lymph nodes on the right side; three of these had simultaneous left cervical lymphadenopathy (9, 10–15). The youngest male reported was only 42 years-of-age (16); the oldest was 87 years-of-age (13). Of the 58 cases, 29 cases received PSA examinations; in each case, the PSA level was abnormal. Of these 29 cases, 26 patients had abnormal PSA levels that exceeded 100 ng/ml.This study not only reported 5 relevant cases, but also systematically summarized the diagnosis, clinical features and prognosis of all 58 patients previously reported in the relevant literature, aiming to improve the knowledge and understanding of these diseases.

The mechanism of metastasis to the supraclavicular lymph nodes remains unclear. A widely accepted theory states that the dissemination of lymph nodes could occur *via* step-wise progression (17). This process first occurs in the regional lymph nodes as obturator and pelvic lymph nodes, then moves through the retroperitoneal lymph nodes, cysterna chyli, and the thoracic duct, where the lymphatic system reunites with the systematic circulation at the left subclavian vein. Left cervical nodes are situated close to the entry of the thoracic duct into the left subclavian vein. Therefore, prostate cancer can spread to the left jugular trunk *via* retrograde spread, thus leading to the enlargement of cervical lymph node on the left side (18).

Metastases to the cervical lymph nodes mostly arise from primary cancer in the head and neck, such as the mucosa of the upper aerodigestive tract and the thyroid glands (8). Therefore, such patients are often first encountered in the head-neck surgery department or the otorhinolaryngology department, rather than urology; this may result in misdiagnosis. From reviewing the relevant literature, eight cases attended departments other than urology on their first hospital visit. These cases were referred from several other departments, including the emergency department (7, 19), oncology department (12, 19), internal medicine (13), thoracic surgery (18) and surgical outpatient clinic (15, 18). All five of our current cases were referred from from thyroid surgery and oncology. By summarizing these specific cases, it was evident that needle biopsy of suspicious masses and PSA immunohistochemistry staining were needed for clinicians to consider the prostate as the suspicious primary tumor site.

Hormonal treatment is still the main option for prostate cancer with clavicle lymph node metastasis. But the prognosis of such cases was generally poor. In the present study, only two patients are still alive, with most (60%) showing a poor response to maximal androgen blockade therapy before rapidly developing CRPC and dying after a short duration, which was similar to the previous study (20). Besides, Jones et al. reported 11 metastatic prostatic carcinoma cases presenting with left-sided cervical lymphadenopathy, the mean survival time was only 29.7 months (21). Surprisingly, Michael et al. reported a patient with metastatic cervical lymphadenopathy who was treated with diethylstilbestrol and survived for 12 years (8). Improving early screening for prostate cancer allows earlier detection and treatment of prostate cancer, which may be extremely important to ameliorate the prognosis of such patients. Previous relevant studies have shown that the occurrence of distant lymph nodes metastases is an independent risk factor for poor prognosis of prostate cancer (22), From the data of the study we obtained, it is clear that all patients were already in a state of distant lymph node metastases at the time of initial diagnosis. Therefore, regular serum PSA tests and urological ultrasonography detection are important way to achieve early screening for prostate cancer. Besides, There are some limitations in this study. First of all, this is a single-center retrospective study, which may not reflect the true incidence rate. Secondly, the mechanism of metastasis to the left cervical lymph nodes is not mentioned in the article.

In conclusion, any elderly male patients presenting with an asymptomatic cervical mass as the initial presentation should be considered the possibility of prostate cancer. Needle biopsy and immunohistochemical staining of lymphatic biopsies, and the determination of serum PSA levels are vital for final diagnosis. The overall prognosis of such cases is generally poor, and hormonal therapy including abiraterone may be beneficial.

## Data Availability

The original contributions presented in the study are included in the article/Supplementary Material, further inquiries can be directed to the corresponding author/s.
